# White shark optimizer with optimal deep learning based effective unmanned aerial vehicles communication and scene classification

**DOI:** 10.1038/s41598-023-50064-w

**Published:** 2023-12-27

**Authors:** T. Nadana Ravishankar, M. Ramprasath, A. Daniel, Shitharth Selvarajan, Priyanga Subbiah, Balamurugan Balusamy

**Affiliations:** 1 Department of Data Science and Business Systems, SRM Institute of Science and Technology, Kattankulathur, Chennai, Tamil Nadu India; 2 Department of Data Science and Business Systems, SRM Institute of Science and Technology, Kattankulathur, Chennai, India; 3 Computer Science & Engineering. Amity School of Engineering and Technology (ASET), Amity University, Gwalior, Madhya Pradesh India; 4 Department of Computer Science, Kebri Dehar University, Kebri Dehar, Ethiopia; 5 School of Built Environment, Engineering and Computing, Leeds Beckett University, Leeds, LS1 3HE UK; 6 Department of Networking and Communications, Faculty of Engineering and Technology, SRM Institute of Science and Technology, Kattankulathur, Chengalpattu District, Tamil Nadu 603203 India; 7 Shiv Nadar University, Delhi NCR, India

**Keywords:** Engineering, Electrical and electronic engineering

## Abstract

Unmanned aerial vehicles (UAVs) become a promising enabler for the next generation of wireless networks with the tremendous growth in electronics and communications. The application of UAV communications comprises messages relying on coverage extension for transmission networks after disasters, Internet of Things (IoT) devices, and dispatching distress messages from the device positioned within the coverage hole to the emergency centre. But there are some problems in enhancing UAV clustering and scene classification using deep learning approaches for enhancing performance. This article presents a new White Shark Optimizer with Optimal Deep Learning based Effective Unmanned Aerial Vehicles Communication and Scene Classification (WSOODL-UAVCSC) technique. UAV clustering and scene categorization present many deep learning challenges in disaster management: scene understanding complexity, data variability and abundance, visual data feature extraction, nonlinear and high-dimensional data, adaptability and generalization, real-time decision making, UAV clustering optimization, sparse and incomplete data. the need to handle complex, high-dimensional data, adapt to changing environments, and make quick, correct decisions in critical situations drives deep learning in UAV clustering and scene categorization. The purpose of the WSOODL-UAVCSC technique is to cluster the UAVs for effective communication and scene classification. The WSO algorithm is utilized for the optimization of the UAV clustering process and enables to accomplish effective communication and interaction in the network. With dynamic adjustment of the clustering, the WSO algorithm improves the performance and robustness of the UAV system. For the scene classification process, the WSOODL-UAVCSC technique involves capsule network (CapsNet) feature extraction, marine predators algorithm (MPA) based hyperparameter tuning, and echo state network (ESN) classification. A wide-ranging simulation analysis was conducted to validate the enriched performance of the WSOODL-UAVCSC approach. Extensive result analysis pointed out the enhanced performance of the WSOODL-UAVCSC method over other existing techniques. The WSOODL-UAVCSC method achieved an accuracy of 99.12%, precision of 97.45%, recall of 98.90%, and F1-score of 98.10% when compared to other existing techniques.

## Introduction

In the last few years, the technology of unmanned aerial vehicles (UAVs) has fascinated in extensive attention as a quickly emerging domain in intellectual research, civil utilization and military applications^1^. UAVs have the benefits of lower cost implementation, scalability smaller size, fast distribution, flexibility, simple access from risky fields and so on. Nevertheless, because of the inadequate energies and computational powers of individual UAVs, it is not possible for ensuring the optimum operational condition at all times^2^, whereas strong connection among the various UAVs to procedure a cluster that could be utilized to achieve different tasks in superior surroundings and complexity^3^. Thus, it progressively becomes a significant form of present applications of UAVs in combat. The UAVs node's superior mobility in FANET (Flying Ad Hoc Network) creates it better frequent for enter and exiting the networks, which can cause complications in the maintenance and establishment of the networks and build it challenging for controlling and managing the UAVs proficiently as their scale raised^4^. Separation of the networks into clusters can support solving the above complications. The separation process is based on dissimilar factors and UAVs are separated into various cluster groups that could be in direct communication with one another and share resources and mediums within the nodes' communication range^5^.

The great performance of the UAV is nominated as Cluster Head (CH) and the other UAVs in the groups are Cluster Members (CM) which can be based on various election considerations^6^. The CH nodes are accountable for inter-and intra-cluster information forwarded in the UAV networks, and then the nodes transmit packets to the CH, which transmits them to the BSs (Base Station) or nodes' destination^7^. Thereby, the control packet will be decreased. Nevertheless, the transmission load of CH can be raised due to it requires to transfer of information between management and also clusters CMs. Consequently, the separation of clusters and the collection of CHs, in addition to the effectiveness of cluster management schemes are crucial for achieving dependable communication and enhancing the network’s performance in a hierarchical network. Once the aerial image scenes are obtained, it endures aerial image classification^8^. By the coverage of different earthed objects, the images are classified into subfields and several lands are covered with dissimilar semantic classes. Therefore, the classification of aerial images is a significant process for many real-time applications namely resource managing, metropolitan planning, RS and also computer cartography^1^. The deep Learning (DL) technique is extremely advantageous in the determination of traditional challenges namely Natural Language Processing (NLP), speech recognition, object detection and then a lot of these kinds of real-time applications. It is vastly more proficient than the standard processes and finally, it is also achieved with more consideration in industries and the scientific community^9^.

This article presents a new White Shark Optimizer with Optimal Deep Learning based Effective Unmanned Aerial Vehicles Communication and Scene Classification (WSOODL-UAVCSC) technique. The WSOODL-UAVCSC technique involves two main components: UAV clustering and scene classification^10^. The WSO algorithm is utilized for the optimization of the UAV clustering process enables to accomplish effective communication and interaction in the network. With dynamic adjustment of the clustering, the WSO algorithm improves the performance and robustness of the UAV system. For the scene classification process, the WSOODL-UAVCSC technique involves capsule network (CapsNet) feature extraction, marine predators algorithm (MPA) based hyperparameter tuning, and echo state network (ESN) classification. A wide-ranging simulation analysis was conducted to validate the enhanced performance of the WSOODL-UAVCSC method.

Unmanned Aerial Vehicles (UAVs) have experienced significant advancements in the fields of electronics and communications, rendering them a highly promising facilitator for the forthcoming era of wireless networks. Unmanned aerial vehicles (UAVs) have demonstrated their versatility and efficacy in a wide range of applications, encompassing intelligent systems such as communication and scene classification. Unmanned Aerial Vehicle (UAV) communication presents novel opportunities for entrepreneurs and innovators to investigate a diverse array of practical applications and transformative solutions^11^. The application of unmanned aerial vehicle (UAV) communications encompasses various scenarios, including the extension of coverage for transmission networks in the aftermath of disasters, facilitating communication for Internet of Things (IoT) devices, and enabling the transmission of distress messages from areas with limited coverage to emergency centers. Nevertheless, the task of improving the clustering of unmanned aerial vehicles (UAVs) and the classification of scenes using deep learning methods continues to pose a significant challenge, as the goal is to attain the highest level of performance. This article introduces a novel approach known as the White Shark Optimizer with Optimal Deep Learning-based Effective Unmanned Aerial Vehicles Communication and Scene Classification (WSOODL-UAVCSC) technique in response to the given context^12^. The main objective of the WSOODL-UAVCSC technique is to facilitate the clustering of Unmanned Aerial Vehicles (UAVs) in order to enhance communication efficiency and optimize scene classification. The WSOODL-UAVCSC technique comprises two primary constituents, namely UAV clustering and scene classification^13^. The WSO algorithm is utilized in the UAV clustering procedure to optimize the configuration of UAV clusters and improve communication and interaction within the network.

The performance and robustness of the UAV system are significantly enhanced by the WSO algorithm through the dynamic adjustment of clustering^14^. The scene classification process implemented by the WSOODL-UAVCSC technique involves multiple stages, namely Capsule Network (CapsNet) feature extraction, hyperparameter optimization using the marine predators algorithm (MPA), and classification utilizing the echo state network (ESN)^15^. The utilization of sophisticated deep learning methodologies significantly enhances the precision and effectiveness of scene classification, thereby enabling unmanned aerial vehicles (UAVs) to make well-informed decisions by leveraging the acquired data. The efficacy of the WSOODL-UAVCSC methodology is verified by means of an extensive simulation analysis. The comprehensive analysis of results demonstrates the superior performance of the WSOODL-UAVCSC method in comparison to existing techniques for clustering Unmanned Aerial Vehicles (UAVs) and classifying scenes^16^. The implementation of the WSOODL-UAVCSC technique has the potential to revolutionize wireless communication networks by leveraging UAVs^17^. This advancement allows for enhanced data transmission, improved scene comprehension, and the facilitation of various innovative applications. The results of this study present novel prospects for enhancing communication and scene classification using unmanned aerial vehicles (UAVs), thereby facilitating progress in the domain of intelligent systems and UAV technology.

The impetus behind the creation of the White Shark Optimizer with Optimal Deep Learning based Effective Unmanned Aerial Vehicles Communication and Scene Classification (WSOODL-UAVCSC) method arises from the increasing potential of Unmanned Aerial Vehicles (UAVs) within the realm of wireless networks and intelligent systems. Unmanned aerial vehicles (UAVs) have emerged as multifunctional instruments for a wide range of applications, encompassing communication and scene classification^18^. This development has created prospects for inventive and transformative solutions. The utilization of Unmanned Aerial Vehicle (UAV) communication presents notable benefits, including the expansion of transmission network coverage in the aftermath of disasters, the facilitation of communication for Internet of Things (IoT) devices, and the prompt dispatching of distress messages from areas lacking coverage to emergency centers. Nevertheless, there exist certain obstacles when it comes to improving the efficacy of UAV clustering and scene classification through the utilization of deep learning methodologies in order to attain the most optimal results^19^.

The WSOODL-UAVCSC technique has been developed to tackle these challenges through the introduction of a novel optimization approach that utilizes the White Shark Optimizer (WSO) for UAV clustering. The primary objective of the technique is to enhance performance and robustness within the network by effectively clustering UAVs, thereby improving communication and interaction^20^. The methodology comprises of two primary elements: Unmanned Aerial Vehicle (UAV) clustering and scene classification. The utilization of the WSO algorithm is employed to optimize the process of clustering Unmanned Aerial Vehicles (UAVs), with the aim of dynamically adjusting the clustering in order to enhance the overall performance of the system. Furthermore, the process of scene classification integrates sophisticated deep learning methodologies, including Capsule Network (CapsNet) for feature extraction, hyperparameter optimization through the marine predators algorithm (MPA), and classification utilizing the echo state network (ESN). The conducted simulation analysis serves to validate the performance of the WSOODL-UAVCSC approach, showcasing its enhanced capabilities in comparison to current techniques^21^. The integration of WSO optimization, feature extraction based on deep learning, and advanced classification techniques yields enhanced outcomes in tasks related to clustering and classification of UAVs and scenes. The primary objective of the WSOODL-UAVCSC technique is to leverage the capabilities of unmanned aerial vehicles (UAVs) in wireless networks and intelligent systems through the optimization of UAV clustering and scene classification procedures^22^. The proposed approach aims to enhance the performance and efficiency of unmanned aerial vehicle (UAV) communication applications, thereby creating opportunities for diverse real-world applications and novel solutions.

Due to advances in electronics and communications, UAVs may enable the next generation of wireless networks. Intelligent systems use UAVs for scene classification and communication. UAV communication enables coverage extension for transmission networks after disasters, Internet of Things (IoT) devices, and sending distress messages from devices in coverage holes to emergency centers. Using deep learning to improve UAV clustering and scene classification is difficult. The White Shark Optimizer with Optimal Deep Learning based Effective Unmanned Aerial Vehicles Communication and Scene Classification (WSOODL-UAVCSC) solves these issues^23^. The WSOODL-UAVCSC method clusters UAVs for communication and scene classification. It includes UAV clustering and scene categorization. The White Shark Optimizer (WSO) method optimizes UAV clustering for network efficiency. WSO dynamically adjusts clustering to improve UAV system performance and reliability. WSOODL-UAVCSC scene classification requires numerous phases. First, CapsNet extracts scene features. The marine predators algorithm (MPA) optimizes CapsNet performance by modifying hyperparameters. Finally, the echo state network (ESN) classifies scenes. A comprehensive simulation investigation validates the proposed approach. The analysis shows that WSOODL-UAVCSC outperforms other methods^24^. The research addresses UAV clustering and scene classification difficulties utilizing deep learning for effective communication and scene analysis. The WSOODL-UAVCSC algorithm improves UAV clustering and scene classification performance.

### Outcomes of the proposed methodology

The WSOODL-UAVCSC disaster management UAV clustering and scene categorization method delivers numerous major results:

1. Complex scene interpretation, data variability, feature extraction from visual data, high-dimensional and nonlinear data, adaptability, real-time decision-making, clustering optimization, and sparse or partial data in UAV clustering and scene classification are addressed.
2. It optimizes UAV clustering and network connectivity via the White Shark Optimizer (WSO) method. Also employed are CapsNet feature extraction, MPA-based hyperparameter tuning, and ESN scene categorization.
3. Numerous simulations proved WSOODL-UAVCSC works. It outperforms existing approaches in accuracy, precision, recall, and F1-score.
4. The WSOODL-UAVCSC method had 99.12% accuracy, 97.45% precision, 98.90% recall, and 98.10% F1-score. These measurements show disaster management UAV clustering and scene categorization methodology's reliability and efficacy.

### Organization of paper

The rest of the paper is structured in the following manner. Section "Related works" e presents a comprehensive examination of the relevant literature and the methodology utilized in this research endeavor. In Section "Proposed methodology", a comprehensive overview of the workflow utilized in the proposed study is provided, along with a detailed explanation of pertinent concepts. The fourth section of the paper is dedicated to the Simulation Setup and Parameters, Performance Metrics, and the comparative analysis of the results obtained. And, finally section "The shot vector is pushed towards and the Long vector is pushed towards by the squashing function." concludes the paper with future scope.

## Related works

Pustokhina et al.,^25^ (2021) presented a new energy-effective cluster-based UAV with a DL-based scene classification (SC) approach. Primarily, the UAVs were clustered utilizing the T2FL approach because of RE, UAV degree, and distance to adjacent UAVs. Afterwards, the selected CHs transfer the captured images to BSs. Second, the DL method-based ResNet_50 system can be exploited for SC. For tuning the hyper-parameters of the ResNet_50 approach, a water wave optimizer (WWO) system can be employed. Finally, the KELM technique was utilized for performing the SC method. Rajagopal et al.^26^, (2020) presented a novel multi-objective PSO (MOPSO) approach for developing recent DCNNs (Deep Convolutional Neural Networks) in SC, which creates the non-dominant solution. This process assists to attain a tradeoff between the inference latency and classification performance, called multi objective convolutional neural network (MOCNN).

Li et al.,^27^ (2018), discussed a new super pixel-based feature was presented in this case to distinguish UAV images. Based on the presented feature, a scene detection approach of the BoW method for aerial imaging was planned. The presented super-pixel-based feature which employs landform data introduces top-task super-pixel extraction of landforms to bottom-task expression of feature vectors. Guo et al.,^28^ (2021), presented an enhanced approach to deep reinforcement learning for unmanned aerial vehicle (UAV) navigation in environments characterized by high levels of dynamism. The proposed methodology demonstrates a higher level of convergence and effectiveness.

Uthayan et al.,^29^ (2022) presented a novel DL-enabled aerial SC approach for UAV-aided MEC methods. The projected method allows the UAVs for capturing aerial images that are transferred to MEC for more processing. A shuffled Shepherd Optimizer (SSO) system was carried out for accomplishing this and to define the hyper-parameters of the CapsNet approach. At last, the BPNN classification approach was executed to define the suitable classes of aerial imagery. Li and Zhou^30^ (2021), the authors deal with scene detection by learning the representation of features automatically in big image instances. Primarily, the authors present a novel system for scene detection using trained a slight-weight CNN (Convolutional Neural Network) which completely takes minimal complex and better network structure and is trainable in the approach of end-to-end. Secondarily, the authors present to use of a salient region-based technique for extracting the local feature representation of certain scene areas directly in the convolutional layer dependent upon the self-selection process, and all the layers apply a linear function with an end-to-end approach.

Xia et al.,^31^ (2021), a novel lightweight method dependent upon VGG16 was presented for extracting various features of RSI by 5 convolutional elements. This method utilizes depthwise separable convolutional for reducing the network limitations. The pooling layer was added for solving the inherent non-adaptive issue of convolutional networks. The global average-pooling layer can be employed to sum the data for making an input spatial transformation further stable.

Ming et al.,^32^ (2021), for scene categorization in UAV remote sensing photos, the research suggested an unsupervised self-adaptive deep learning classification network. Both the Attention U-Net and the Mask RCNN performed well in classification when it came to describing finer details. Classification networks based on unsupervised adaptive learning are used both for classification and Sample retrieval strategy that automatically adjusts to homology and reliability.

Nilakshi and Bhogeswar^33^ (2021), the study presented a novel methodology for feature selection in aerial scene classification, utilizing mutual information as the basis for efficient transfer learning. The presented study introduced an innovative approach for feature selection, utilizing mutual information as the primary criterion and enhanced transfer learning in the domain of aerial scene classification.

Yu et al.,^34^ (2021), presented on development of a guidance algorithm based on deep reinforcement learning, specifically designed for collision avoidance in fixed-wing unmanned aerial vehicles (UAVs). The research does not address aspects related to communication or scene classification. This paper introduced a computational guidance method for collision avoidance in limited airspace for multiple fixed-wing UAVs, utilizing deep reinforcement learning techniques. The algorithm under consideration demonstrated a high level of efficacy in mitigating the likelihood of collisions among multiple unmanned aerial vehicles (UAVs), even when the number of aircraft involved is substantial. The application of deep reinforcement learning in the context of collision avoidance. The presented study aims to explore an extension of the actor-critic model within the context of reinforcement learning.

**Table:** 

Paper	Methodology	Contribution
Sarfraz, Ahmed, Dakhan^50^ (2022)	The suggested approach for ensemble learning, which utilises multiple objective particle swarm optimisation, demonstrates enhancements in subject-independent emotion identification based on EEG data	The present study introduces a novel ensemble learning approach that demonstrates superior recognition performance compared to previous methodologies
Omurkanova^51^ (2022)	This study presented a novel computer-based diagnostic model for the diagnosis of brain tumours. The model incorporates textural feature extraction algorithms, convolutional neural network features, and optimization algorithms. The accuracy rate of the model is 98.22%	The present study introduced a novel computer-based hybrid diagnostic model and employs optimisation methods for the purpose of feature selection
Mohammad-Hossein, Nadimi-Shahraki et al.^52^ (2022)	This work presented a novel approach, namely the Enhanced Whale Optimisation method (E-WOA), for the purpose of medical feature selection. The proposed method is applied to a case study involving the identification of relevant features in the context of COVID-19. The E-WOA algorithm has superior performance compared to other variations and exhibits efficiency in the selection of effective characteristics	The present study introduced an improved version of the whale optimisation algorithm, referred to as the enhanced whale optimisation algorithm (E-WOA). Specifically, a binary variant of the E-WOA, known as the binary enhanced whale optimization algorithm (BE-WOA), is proposed for the purpose of medical feature selection
Kappelhof et al.^53^ (2021)	This study presented an innovative evolutionary algorithm designed for the purpose of reliably predicting unfavourable outcomes following endovascular treatment for acute ischemic stroke, specifically focusing on the application of fuzzy decision trees	This work introduced a fuzzy decision tree-based evolutionary method to consistently predict poor outcomes after endovascular treatment for acute ischemic stroke
Javier, Enrique, et al. (2021)	The practical application of robust multimodal registration of fluorescein angiography (FA) and optical coherence tomography angiography (OCTA) images has garnered growing attention. The simultaneous examination of fundus autofluorescence (FA) and optical coherence tomography angiography (OCTA) pictures provide shared and supplementary visual data that can be utilised in the diagnosis and classification of retinal diseases	Clinical practice increasingly seeks robust multimodal registration of fluorescein and OCTA pictures. Combining FA and OCTA images gives complementing visual information for detecting and grading retinal diseases
Yu et al.^35^ (2020)	Utilized reinforcement learning to address collision avoidance and optimal trajectory planning in UAV communication networks	Introduced a reinforcement learning methodology for collision avoidance and trajectory planning in UAV communication networks
Oualid and Deok^36^ (2021)	Employed actor-critic-based reinforcement learning for autonomous navigation and collision prevention in unfamiliar outdoor settings	Developed a system enabling autonomous navigation and collision prevention in unfamiliar outdoor settings using reinforcement learning techniques
Chao et al.^37^ (2020)	Proposed the LwH algorithm integrating deep reinforcement learning for UAV navigation in complex environments with sparse rewards	Introduced the LwH algorithm, utilizing deep reinforcement learning and assistance from non-experts for UAV navigation in sparse reward environments
Chi et al.^38^ (2020)	Presented a decentralized deep reinforcement learning framework for efficient multi-UAV navigation and energy minimization	Introduced a decentralized deep reinforcement learning framework for multi-UAV navigation and energy management, outperforming existing approaches
Carlos et al.^39^ (2019)	Explored deep learning models for object classification and reinforcement learning techniques for UAVs in indoor environments with obstructions	Investigated deep learning for object classification and reinforcement learning for UAVs, validating efficacy in indoor environments with obstacles
Hang et al.^40^ (2020)	Proposed the UC-DDPG algorithm based on deep reinforcement learning to optimize energy efficiency and fairness in 3D UAV control within wireless systems	Introduced the UC-DDPG algorithm for energy-efficient and fair 3D UAV control, showing superior performance compared to alternative scheduling methods
Sana et al.^41^ (2021)	Explored machine learning solutions for UAV communication and resource management, without a focus on deep learning or scene classification	Investigated machine learning-based solutions for air-to-air, air-to-ground, and ground-to-air UAV communication and resource management
Jiseon et al.^42^ (2021)	Utilized deep reinforcement learning for precise target tracking and management of multiple UAVs, ensuring high accuracy and low runtime costs	Employed deep reinforcement learning for precise target tracking and multi-UAV control, achieving high accuracy with low runtime costs
Chao et al.,^43^ (2022)	Explored deep reinforcement learning for collision-free flocking of fixed-wing UAVs, excluding communication and scene classification aspects	Developed the MA2D3QN algorithm for collision-free flocking in fixed-wing UAVs, demonstrating scalability and adaptability in simulation environments
Omar et al.^17^ (2021)	Investigated the use of UAVs for emergency and rescue operations, focusing on guidance without delving into communication or scene classification	Studied the utilization of UAVs for emergency vehicle guidance and intervention strategies in rescue operations

The research uncovered numerous cutting-edge methods, including unmanned aerial vehicles (UAVs), deep learning, scene classification, and reinforcement learning, among others. However, a significant technical void exists in the integration of multiple methods to comprehensively address complex real-world circumstances. Although a number of studies have focused on features such as energy-efficient clustering, scene classification, and collision avoidance, there has been surprisingly little research into comprehensive solutions that incorporate all of these elements. The lack of cohesive frameworks that integrate advanced approaches for tasks such as autonomous navigation, communication optimisation, and dynamic scene interpretation is one of the obstacles that must be surmounted in order to achieve efficient and adaptable UAV operations. In addition, standardised evaluation criteria and benchmark datasets are still required to facilitate the effective comparison and validation of proposed approaches, despite the progress made in certain fields.

To bridge this technical chasm, a concerted effort towards the development of integrated, multifaceted solutions that capitalise on the strengths of each approach is required. These solutions must efficiently manage the complexities of UAV applications in the actual world.A variety of innovative methodologies involving UAVs, deep learning, scene classification, and reinforcement learning emerged from the research survey. However, a significant technical void exists in the integration of these approaches to comprehensively address complex real-world scenarios. Despite the fact that a number of studies have focused on particular aspects such as energy-efficient aggregation, scene classification, and collision avoidance, there has been limited investigation into holistic solutions that combine these elements. The absence of cohesive frameworks integrating advanced techniques for tasks such as autonomous navigation, communication optimisation, and dynamic scene comprehension is a barrier to achieving seamless and adaptable UAV operations. In addition, despite the progress made in individual disciplines, there is a need for more standardised evaluation metrics and benchmark datasets to facilitate the comparison and validation of proposed methodologies. Closing this technical gap requires a concerted effort to develop integrated, multi-faceted solutions that leverage the assets of each approach to effectively address the complexities of UAV applications in the real world.

## Proposed methodology

**Figure 1:**
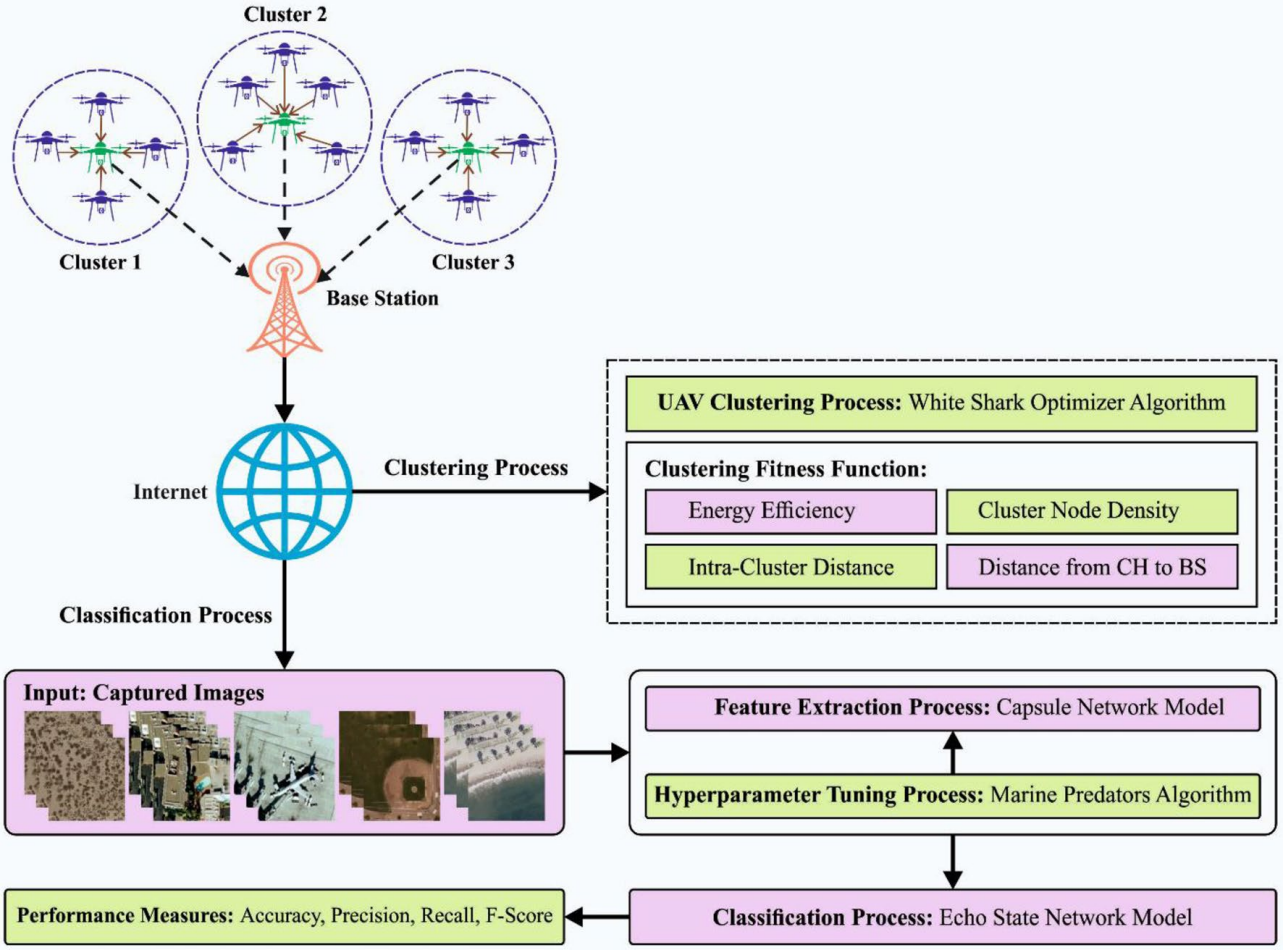
Overall process of WSOODL-UAVCSC methodology.

In this article, we have focused on the development of the WSOODL-UAVCSC for effective transmission and scene classification in the UAV network. The major aim of the WSOODL-UAVCSC technique is to cluster the UAVs for efficient communication and scene classification. The WSOODL-UAVCSC technique involves two main components: UAV clustering and scene classification. Figure 1 depicts the overall procedure of the WSOODL-UAVCSC method. The WSOODL-UAVCSC methodology is a comprehensive framework that has been developed to tackle the issues associated with communication and scene classification in Unmanned Aerial Vehicle (UAV) systems. This methodology takes a multi-faceted approach to address these challenges. The present methodology incorporates a range of sophisticated methodologies and algorithms in order to optimise the effectiveness of unmanned aerial vehicle (UAV) networks during disaster response situations.

The WSOODL-UAVCSC framework encompasses a series of distinct stages:

The methodology commences with the application of the White Shark Optimizer (WSO) algorithm, which facilitates the optimisation of Unmanned Aerial Vehicles (UAVs) clustering. The technique exhibits dynamic properties by adapting the clustering process to optimise communication and interaction within the network. The objective is to optimise performance and resilience, which are of utmost importance in situations of catastrophic events.

The WSOODL-UAVCSC framework utilises Capsule Networks (CapsNet) for the purpose of feature extraction. This is subsequently followed by the application of the Marine Predators Algorithm (MPA) to perform hyperparameter tuning. Finally, the Echo State Network (ESN) is employed for scene categorization. The objective of this multi-layered deep learning methodology is to effectively categorise situations that have been recorded by unmanned aerial vehicles (UAVs), which is a crucial component in the field of disaster management.

### System model

#### Phase I: clustering process using the WSO algorithm

The WSO algorithm is utilized for the optimization of the UAV clustering process and enables to accomplish effective communication and interaction in the network. With dynamic adjustment of the clustering, the WSO algorithm improves the performance and robustness of the UAV system.

The maximum speed of a UAV reaches up to $30 m/s$. All the UAV devices are based on the location‐aware module which enables the routing technique to be an efficient and precise function. Generally, position data was obtained from the alternate system. In this work, GPS and inertial measurement units are provided for the deployment and motion sensing of UAVs. Every UAV is aware of its BSs and neighbours' location. All UAVs are equipped with short and long-range wireless transmissions. For intra‐transmission, short-range wireless transmission is applied with the peers in the cluster. For inter‐cluster transmission, long-range wireless transmission is applied with its BSs and other CHs.

#### Design of WSO algorithm

WSO is a metaheuristic optimization approach affected by the attributes of white sharks namely their sense of smell while foraging and navigating and their exceptional hearing^44^. The steps for the WSO algorithm are given as follows:

Movement speed toward prey. Once a white shark identifies the prey position based on the waves generated by the activities of the target:

1 $${s}_{i}(t+1)=u\left[{s}_{i}\left(t\right)+{\rho }_{1}\cdot {c}_{1}\left({P}_{Gbest}\left(t\right)-{P}_{i}\left(t\right)\right)+{\rho }_{2}\cdot {c}_{2}\left({P}_{ibest}\left(t\right)-{P}_{i}\left(t\right)\right)\right]$$

In Eq. (1), the index $i(i=\mathrm{1,2}, \dots , n)$ formulates the white shark command in the population of size $n,$ $s$ signifies the speed, $p$ shows the current location vector of ${i}^{th}$ white sharks, ${P}_{gbest}$ shows the high strategic standing vector, ${P}_{best}$ indicates the present optimum location obtained so far, ${c}_{1}$ and ${c}_{2}$ are two random numbers between $[\mathrm{0,1}],$ ${p}_{1},$ ${p}_{2}$, and $u$ are evaluated by using Eqs. (2), (3), and (4):

2 $${\rho }_{1}={\rho }_{max}+\left({\rho }_{max}-{\rho }_{min}\right){e}^{-(4t/{t}_{max}{)}^{2}}$$

3 $${\rho }_{2}={\rho }_{max}+\left({\rho }_{max}-{\rho }_{min}\right){e}^{-(4t/{t}_{max}{)}^{2}}$$

4 $$u=\frac{2}{\left|2-\tau -\sqrt{{\tau }^{2}-4\tau }\right|};\tau =4.125$$

The movement towards optimal prey: once they smell the fragrance of the target or see the prey movement or they presumably identify the waves caused by the prey movement, white sharks continuously travel towards the prey. The prey either leaves or escapes its position to find food. But still, there is the fragrance in that location. Consequently, the position was updated by the white shark:

5 $${P}_{i}\left(t+1\right)=\{{P}_{i}\left(t\right)\neg \oplus {P}_{0}+high\cdot a+low\cdot b; rand<m {P}_{i}\left(t\right)+{s}_{i}\left(t\right)/f; rand\ge m$$

In Eq. (5), $a$ and $b$ represent a 1D binary vector,$high$ and $low$ denotes the upper and lower random search bounds, $f$ refers to the frequency of the wave movement, and $mv$ can be defined as follows:

6 $$m={\left(\left|{a}_{0}+{e}^{\frac{{t}_{max/2}-t}{{a}_{1}}}\right|\right)}^{-1}$$

Let ${a}_{0}$ and ${a}_{1}$ be the two constant parameters.

The movement towards the white shark: The formula for this phase is provided as follows:

7 $$m={\left(\left|{a}_{0}+{e}^{\frac{{t}_{max/2}-t}{{a}_{1}}}\right|\right)}^{-1}$$

8 $${P}_{i}\left(t+1\right)=\{{P}_{Gbest}\left(t\right)+{r}_{1}\cdot D\cdot sgn\left({r}_{2}-0.5\right); {r}_{3}<{s}_{s} {P}_{i}\left(t\right); otherwise$$

where ${r}_{1},$ ${r}_{2}$, and ${r}_{3}$ represent the random value ranges within $[\mathrm{0,1}]$, and $D$ shows the distance between the targets and the sharks.

Fish school behaviours: this phase was modelled by Eq. (9):

9 $${P}_{i}\left(t+1\right)=\frac{{P}_{i}\left(t\right)+{P}_{i}\left(t+1\right)}{2\cdot rand}$$

#### Process involved in clustering technique

The WSOODL-UAVCSC method measures a fitness function by adding various parameters. The WSOODL-UAVCSC technique is developed with the existence of four fitness parameters such as UAV nodes, average distance of UAVs for CHs enclosed by the sensing range, distance in CH to sink, and energy efficiency of cluster node density^45^. The data on fitness parameter was shown as follows:

Energy efficiency: The CH performs diverse activities namely sense, gathered, aggregation, data broadcast, etc.; thus, when compared to other nodes, CH intakes a considerable amount of energy. Next, it is essential to determine an FF that shared the load amongst UAVs from the network:

$${R}_{e}=e\left({n}_{i}\right)$$

$$A\nu {g}_{e}=\frac{1}{n}{\sum }_{i=0}^{n}e\left({n}_{i}\right)$$

10 $${f}_{1}=C{H}_{opt}*\frac{{R}_{e}}{Av{g}_{e}}=\frac{C{H}_{opt}*e\left({n}_{i}\right)}{\frac{1}{n}{\Sigma }_{i=0}^{n}e\left({n}_{i}\right)}\forall C{H}_{opt}=5\% of n,e\left(n\right)=0.5J or 1.25J or 1.75J$$

In Eq. (10), $C{H}_{opt}$ indicates the optimal percentage of CHs, ${R}_{e},$ $A\nu {g}_{e}$, and ${n}_{i}$ indicate the node $RE$, the average energy of the network, and the overall amount of nodes in UAV, correspondingly.

Cluster node density: the cost is a key parameter for the higher energy efficacy of the network During intra‐cluster transmission. As soon as the cost function of the cluster was defined, then the deployment of network energy becomes larger as follows:

11 $${f}_{2}=max\left(n\left(C{H}_{1}\right),n\left(C{H}_{2}\right),n\left(C{H}_{3}\right)n\left(C{H}_{j}\right)\right)\forall n=2 To 95, j=1 to 15$$

where $n\left(C{H}_{j}\right)$ indicates the quantity of UAVs from the range of $\left(C{H}_{j}\right)$ the $\left(C{H}_{j}\right)$. The value of objective function ${f}_{2}$ is better than the effective selection of CH and exploited from the energy deduction.

The average distance of UAV to the CHs within the sensing range: In intra-cluster transmission, UAV transmits data to the CH. The energy of UAV reduces, once the CH is far away from the CM; there is a deployment of low energy afterwards the CHs is nearer to the member UAV nodes,

12 $${f}_{3}=\frac{1}{{n}_{s\tau }}{\sum }_{i=0}^{{n}_{sr}}disT\left(CH, i\right) \forall dist\left(CH, i\right)=1 to 35 m,{n}_{sr}=1 to 100$$

In Eq. (12), ${n}_{sr}$ and $dist$ $(CH, i)$ show the amount of $CH$ from the sensing sequence of the cluster and UAVs from the sensing range and Euclidean distance in nodes. Therefore, the value of ${f}_{3}$ is minimal; but, the intra‐cluster transmission energy can be declined.

Distance from CH to BS: The distance between CHs and BSs takes a crucial function as if the $CHS$ is distant from the sink and quickly exploits energy as follows:

13 $${f}_{4}=\frac{1}{CH}{\sum }_{i=0}^{CH}dist\left(BS, C{H}_{i}\right) \forall dist\left(BS, C{H}_{i}\right)=1 to 70m, CH=1 to 15$$

In Eq. (13), $dist(BS, cH)$ shows the Euclidean distance between $C{H}_{i}$ and $BS$. Minimizing the ${f}_{4}$ objective function displays that the CHs are not far from BSs. Once the ${f}_{1},{f}_{2},{f}_{3}$, and ${f}_{4}$ parameter functions are calculated, then the objective function is called FF and evaluated by Eq. (14):

14 $$F=Maximize Fitness=\alpha *{f}_{1}+\beta *{f}_{2}+\gamma *\frac{1}{{f}_{3}}+\delta *\frac{1}{{f}_{4}}$$

where $\alpha ,\beta ,\gamma$, and $\delta$ correspondingly indicate the weight coefficient for ${f}_{1},{f}_{2},{f}_{3}$, and ${f}_{4}$ FF parameters, The weight coefficient ranges between [$\mathrm{0,1}$].

### Architecture and working

#### Phase II: scene classification process

For the scene classification process, the WSOODL-UAVCSC technique involves CapsNet feature extraction, marine predators algorithm (MPA) based hyperparameter tuning, and ESN classification.

#### CapsNet feature extraction

The CapsNet model is used for extracting features from the images. CapsNet (the capsule network) uses vector‐wise” encoding, where items are encoded by capsules (collections of neurons). It assists to fix the location of objects and manage the relationship between them^46^. It resolves the problems of information loss caused by the pooling layer in CNN namely scale, location, size, and rotation.

**Figure 2:**
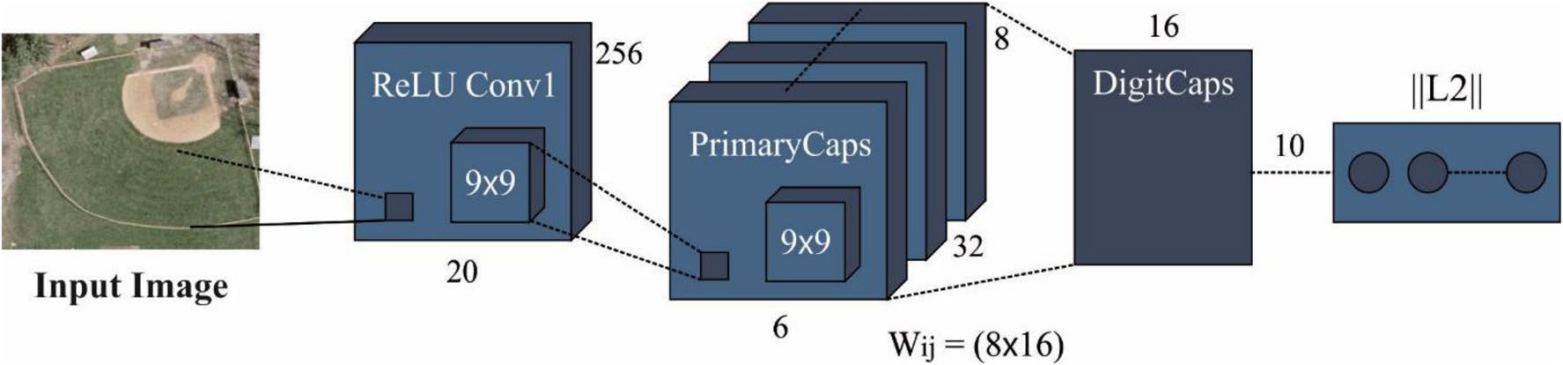
Architecture of CapsNet.

A capsule is composed of a matrix or pose vector for encoding the object's instantiation of activation and different layers parameters. The instantiation parameter changes as the viewing circumstance change, however, the capsule remain active. With the capability of assigning parts to wholes, invariance, and equivariance are two qualities that are used to construct visual hierarchical connections. Figure 2 illustrates the infrastructure of CapsNet.

CapsNet simulates visual hierarchical relationships due to the “Dynamic routing” technique. In CapsNet, dynamic routing is used for establishing visual hierarchical relationships through the technique named "routing‐by‐agreement" to repeatedly route data transition from low to high-level capsules that is the central idea of dynamic routing in CapsNet.

Initially, the ReLU function is activated with 256 filters and takes the parameter of size $9$×$9$ with a stride of 1. The feature was passed to the primary capsule through this function. CapsNet involves three different mechanisms:

- Squash function,
- Convolution, and
- Reshaping function.

The input is provided to the convolutional layer during the convolution process for generating a list of “feature maps”. Here, this feature map was reshaped by the Reshaping function. At last, the entire vector’s length is kept inside the range of $0$ and $1$, based on the squash function. Because it signifies the probability that an item will be found at a particular place in the image and it does not cause the positional data contained in a high dimensional vector to be destroyed $.$

Consider that $l$ and $l+1$ layers have $m$ and $n$ capsules, correspondingly. The activation of the capsules at $the l+1$ layer was computed based on the activation at the $l$ layer. The letter $u$ represents capsule activations at $the l$ layer. We should evaluate $v$, the capsule activation, at $the l+1$ layer $.$

For a ${j}^{th}$ capsule at $l+1$ layers $.$

1. At the $l$ layer, the capsule was used to evaluate the prediction vector. The prediction vector for ${j}^{th}$ capsule ($l+1$ layer) produced by ${i}^{th}$ capsules ($l$ layer) is:
  15 $${u}_{j|i}={W}_{ij}{u}_{i}$$
  In Eq. (15), ${W}_{ij}$ is the weight matrix.
2. Here is the output vector for ${the j}^{th}$ capsules that are evaluated. The output vector for ${the j}^{th}$ capsule is the sum of the weight of each prediction vector supplied by $l$ layer capsules:
  16 $${s}_{j}={\sum }_{i=1}^{m}{c}_{ij}{u}_{j|i}$$
3. Scalar ${c}_{ij}$ signifies the coupling coefficient between capsules $i$ ($l$ layer) and $j$ ($l+1$ layer). The technique named iterative dynamic routing technique defines this coefficient.
4. The squashing function is used to the output vector for obtaining ${v}_{j}$ activation of the ${j}^{th}$ capsule:
  17 $${v}_{j}=squash\left({s}_{j}\right)$$
5. The shot vector is pushed towards $0$ and the Long vector is pushed towards $1$ by the squashing function.

#### Hyperparameter tuning

For adjusting the hyperparameters related to the CapsNet model, the MPA is used. MPA is a bio-inspired metaheuristic technique proposed to overcome complex optimization problems by using biological processes and natural events^47^. The foraging strategy of marine predators in the wild serves as a basis for the mathematical modelling of MPA. MPA accommodates the Brownian statistical and Lévy distributions. The Brownian technique makes the consistent and systematic progression through the search space, whereas The Lévy search method includes traversing space with the sequence of prominent hops. The Brownian search process guarantees visit to remote places. This phenomenon has drastically improved the search abilities of MPA.

In the MPA method, the movement equation is the most important. It directs how the predator moves around the solution space. This can be formulated as follows:

18 $${X}_{i}\left(t+1\right)={X}_{i}\left(t\right)+{v}_{i}\left(t\right)$$

In Eq. (18), ${x}_{i}(t)$ shows the position of the ${i}^{th}$ predator at $t$ time $,{ v}_{i}(t)$ indicates the velocity of the ${i}^{th}$ predators at $t$ time, and $t$ shows the existing iteration of the model.

The MPA's strength lies in its adaptability to multi-modal and fast convergence to optimum solutions and massively parallel optimization problems. The technique requires parameter tuning and might be stuck in the local optima.

The MPA method not only derives a fitness function to attain higher efficiency of classification and also describes a positive integer to represent the better outcome of the solution candidate. The decline of the classification error rate is considered a fitness function.

$$fitness\left({x}_{i}\right)=ClassifierErrorRate\left({x}_{i}\right)$$

19 $$=\frac{number of misclassified samples}{Total number of samples}*100$$

#### Image classification

Finally, the ESN model classifies the input images into distinct class labels. ESN comprises 3 layers such as output, reserve, and input layers. Since the weighted matrix of the input layer and internal connection matrix of the reserve pool (RP) can be arbitrarily created and set, the computational count of trained methods is decreased^48^.

The ESN resolves the fitting regression time sequence problems by exchanging the FC hidden state with spare connection RP; the upgrade layer of the network together with the resultant formula as:

20 $$x\left(t\right)=\left(1-a\right)x+a\cdot tanh\left(Rx\left(t-1\right)+Wu\left(t\right)\right)$$

21 $$y\left(t\right)={W}_{out}x\left(t\right)$$

whereas $tanh$ denotes the activation function and is utilized for obtaining the network echo features, $a$ denotes the rate of leakage utilized for controlling the upgrade weighted of ESN network, ${W}_{in}$ stands for the matrix of input weighted arbitrarily created in the range of 1 and 1, $R$ implies the connection matrix with sparse design inside the RP, $u(t)$ defines the input at time $t,$ $x(t)$ stands for the $t$‐moment layer of the RP, and $y(t)$ indicates the outcome at time $t$. The resultant matrix ${W}_{oui}$ of the ESN is resolved using ridge regression with the subsequent optimizer objectives:

22 $$min \| {W}_{out}X-Y{\| }_{2}^{2}+\lambda \| {W}_{out}{\| }_{2}^{2}$$

23 $${W}_{out}=Y{X}^{T}(X{X}^{T}+\lambda I{)}^{-1}$$

whereas, $\lambda$ stands for the regularized co-efficient utilized for preventing over-fitting in the ESN-trained set, and $I$ represent the identity matrix. The forecast data can be replaced as Eqs. (20) and (21) to acquire the last forecast outcome.

The ESN design is easy and practical; but its forecast outcome was affected by parameter settings, like the RP connection matrix scaling parameter represented by ${R}_{h}$, $N$ denotes the count of RP network nodes, ${I}_{S}$ denotes the input data scaling co-efficient, $S$ implies the RP sparsity degree, and $a$ refers to the leakage value. Employing suitable parameter settings efficiently improves the forecast ability of the ESN.

## Experimentation, results and discussion

### Simulation setup and parameters

- Number of UAVs (n): 10
- UAV Mobility Model: Random Waypoint Model
- UAV Speed: 10 m/s
- Communication Range (Rc): 200 m
- Base Station (BS): Located at coordinates (0, 0) for centralized data processing.

#### For clustering

- Population Size: 50
- Maximum Iterations (MaxGen): 100

#### Hyperparameters

- Learning Rate: 0.001
- Batch Size: 32
- Number of Epochs: 50

### Performance metrics

#### Accuracy

The accuracy of a classification model is determined by calculating the proportion of correctly predicted instances, which includes both true positives and true negatives, relative to the total number of instances present in the dataset. From a mathematical standpoint, it can be formulated as follows:

$$Accuracy= \frac{TP+TN}{TP+TN+ FP+FN}$$

#### Precision

Precision is a metric that serves as an indicator of the performance of a machine learning model. It specifically measures the quality of positive predictions made by the model. Precision is a metric that quantifies the proportion of accurate positive predictions in relation to the total number of positive predictions. It is calculated by dividing the number of true positives by the sum of true positives and false positives.

$$Precision= \frac{TP}{TP+FP}$$

#### Recall

The recall metric is determined by dividing the number of correctly classified Positive samples by the total number of Positive samples. The recall metric quantifies the model's capacity to accurately identify positive samples. There is a positive correlation between recall and the number of positive samples detected.

$$Recall= \frac{TP}{TP+FN}$$

#### F1-score

The F1 score can be defined as the harmonic mean of precision and recall, thereby offering a well-balanced evaluation of the model's efficacy by incorporating both metrics. Precision is a metric that quantifies the ratio of accurately predicted positive instances (true positives) to the total number of positive predictions made by the model. In contrast, recall quantifies the ratio of correctly identified positive predictions to the total number of positive instances present in the dataset.

$$F1-score= \frac{2 * \left(precision * recall\right)}{precision+ recall}$$

### Result analysis

In this section, the clustering and scene classification outcomes of the WSOODL-UAVCSC technique are examined. The scene classification results of the WSOODL-UAVCSC technique are tested on the UCM dataset^49^. This is a 21-class land use image dataset with 100 images of each class. Each image measures 256 × 256 pixels.

**Figure 3:**
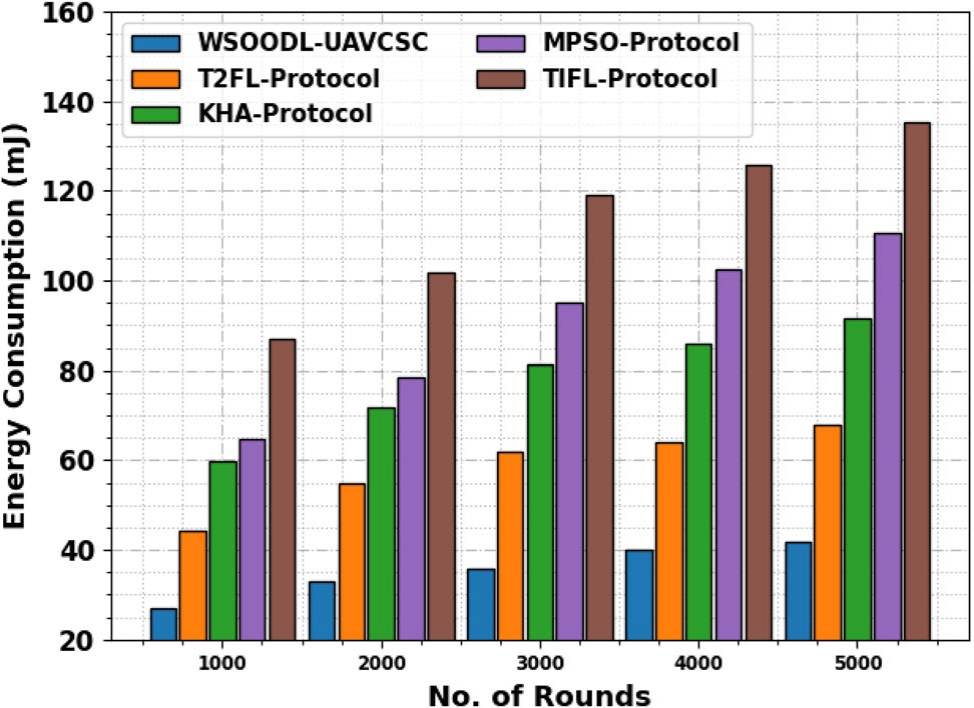
ECOM outcome of WSOODL-UAVCSC system on varying rounds.

Table 1 and Fig. 3 exhibits the energy consumption (ECOM) outcomes of the WSOODL-UAVCSC technique with present techniques. The results show that the TIFL model shows worse outcomes with maximum ECOM values. At the same time, the KHA and MPSO models obtain slightly boosted performance with moderate ECOM values. Although the T2FL model illustrates considerable performance, the WSOODL-UAVCSC technique demonstrates superior results with the least values of ECOM.

**Table 1:** ECOM outcome of WSOODL-UAVCSC system with other methods on varying rounds.

Energy consumption (mJ)					
No. of rounds	WSOODL-UAVCSC	T2FL-protocol	KHA-protocol	MPSO-protocol	TIFL-protocol
1000	26.81	44.09	59.68	64.76	86.79
2000	32.91	54.93	71.88	78.31	101.70
3000	35.96	62.05	81.36	94.92	118.98
4000	40.02	64.08	85.77	102.37	125.75
5000	41.72	67.81	91.53	110.51	135.24

**Figure 4:**
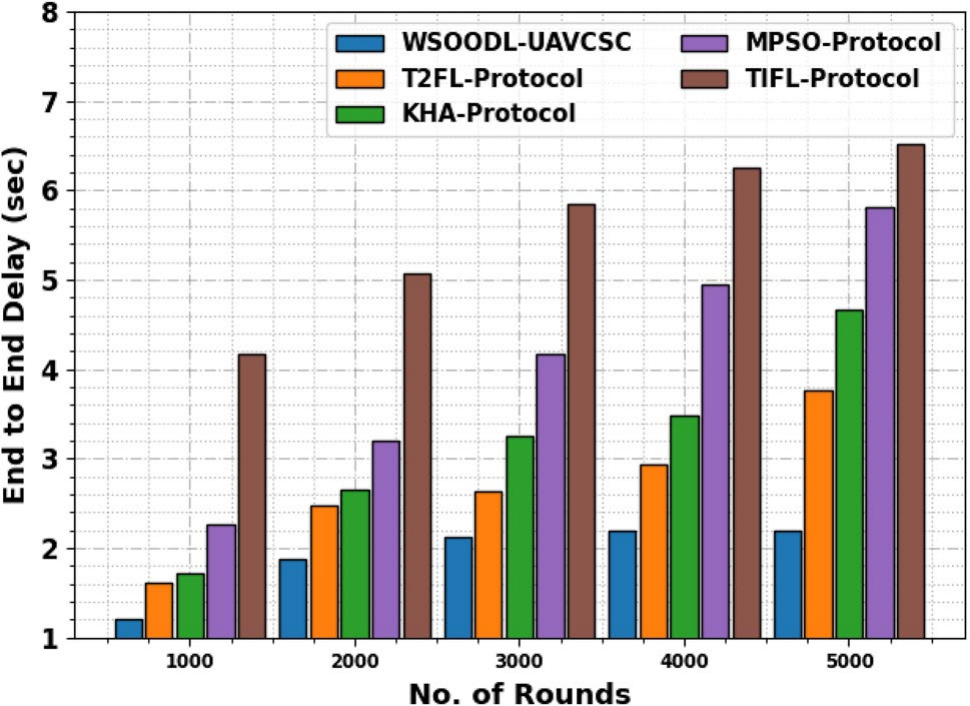
ETED outcome of WSOODL-UAVCSC system on varying rounds.

Table 2 and Fig. 4 show the end-to-end delay (ETED) effects of the WSOODL-UAVCSC approach with present systems. The outcomes exposed that the TIFL method demonstrates worse results with maximal ETED values. Simultaneously, the KHA and MPSO methods acquired moderately increased performance with enough ETED values. Though the T2FL system demonstrates significant performance, the WSOODL-UAVCSC method exhibits greater outcomes with minimum values of ETED.

**Table 2:** ETED outcome of WSOODL-UAVCSC system with other methods on varying rounds.

End-to-End Delay (sec)					
No. of rounds	WSOODL-UAVCSC	T2FL-protocol	KHA-protocol	MPSO-protocol	TIFL-protocol
1000	1.21	1.62	1.72	2.26	4.17
2000	1.87	2.48	2.65	3.20	5.07
3000	2.13	2.63	3.26	4.17	5.85
4000	2.20	2.94	3.49	4.95	6.26
5000	2.20	3.76	4.66	5.81	6.51

**Figure 5:**
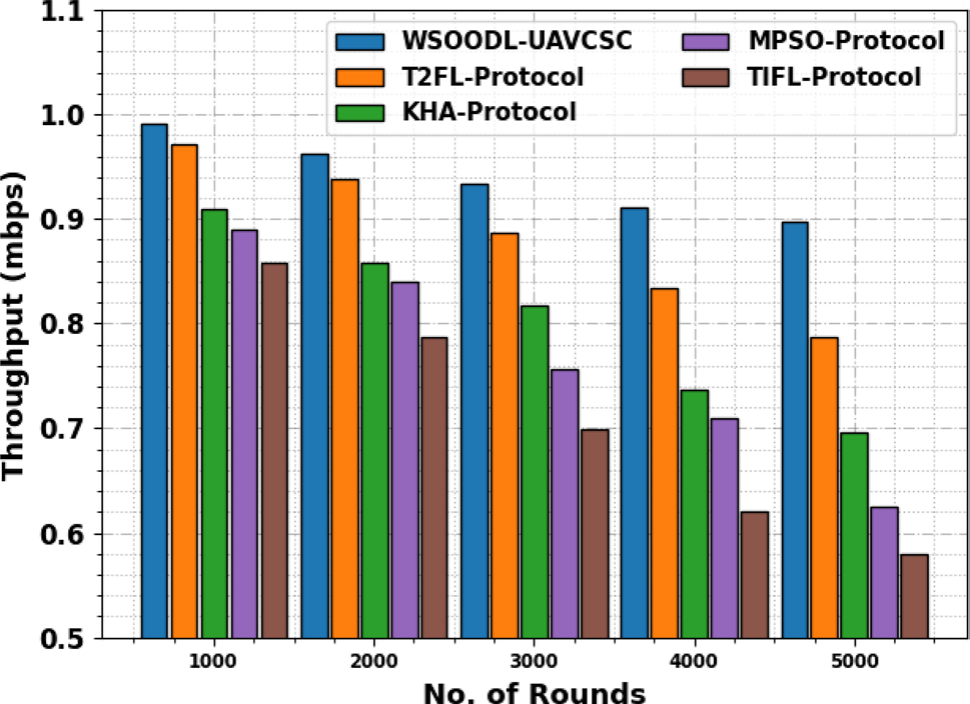
TRHT outcome of WSOODL-UAVCSC system on varying rounds.

In Table 3 and Fig. 5, the throughput (TRHT) outcomes of the WSOODL-UAVCSC technique are compared with existing approaches under varying rounds. The resultant values indicate that the WSOODL-UAVCSC technique reaches increased values of TRHT. For example, on 1000 rounds, the WSOODL-UAVCSC method attained an increased TRHT of 0.99Mbps while the T2FL, KHA, MPSO, and TIFL models offered reduced THRT of 0.97Mbps, 0.91Mbps, 0.89Mbps, and 0.86Mbps correspondingly. Moreover, on 5000 rounds, the WSOODL-UAVCSC system reached to increase TRHT of 0.90Mbps but the T2FL, KHA, MPSO, and TIFL techniques provided decreased THRT of 0.79Mbps, 0.70Mbps, 0.63Mbps, and 0.58Mbps correspondingly.

**Table 3:** TRHT outcome of WSOODL-UAVCSC system with other methods on varying rounds.

Throughput (mbps)					
No. of rounds	WSOODL-UAVCSC	T2FL-protocol	KHA-protocol	MPSO-protocol	TIFL-protocol
1000	0.99	0.97	0.91	0.89	0.86
2000	0.96	0.94	0.86	0.84	0.79
3000	0.93	0.89	0.82	0.76	0.70
4000	0.91	0.83	0.74	0.71	0.62
5000	0.90	0.79	0.70	0.63	0.58

**Figure 6:**
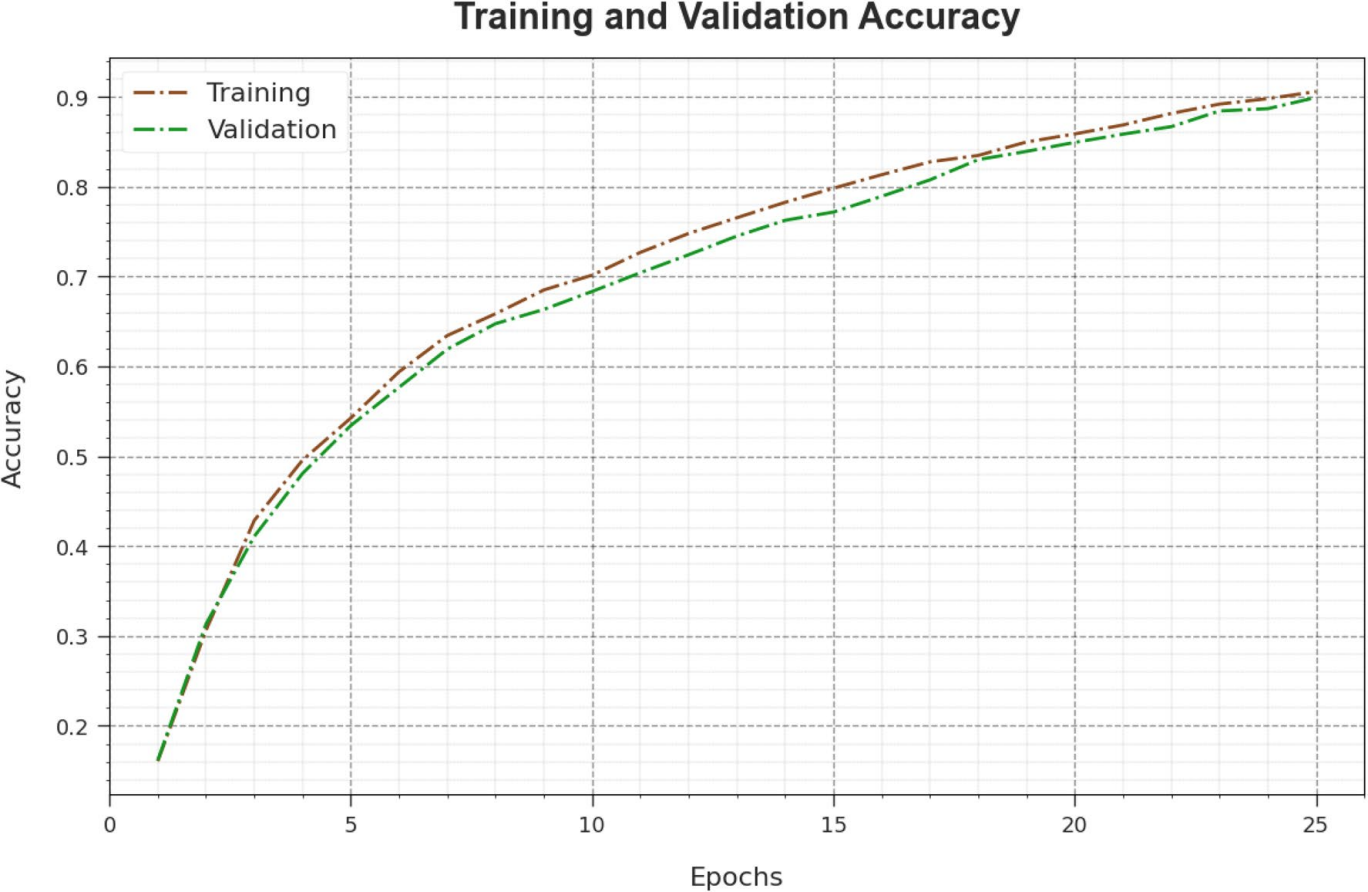
$Acc{u}_{y}$ curve of the WSOODL-UAVCSC system.

Figure 6 shows the training accuracy $TR\_acc{u}_{y}$ and $VL\_acc{u}_{y}$ of the WSOODL-UAVCSC approach. The $TL\_acc{u}_{y}$ is described by the estimation of the WSOODL-UAVCSC system on the TR database however the $VL\_acc{u}_{y}$ is computed by calculating the performance on an individual testing database. The outcomes demonstrated that $TR\_acc{u}_{y}$ and $VL\_acc{u}_{y}$ raising with an upsurge in epochs. Accordingly, the performance of the WSOODL-UAVCSC systems acquires to enhance the TR and TS database with an increase in many epochs.

**Figure 7:**
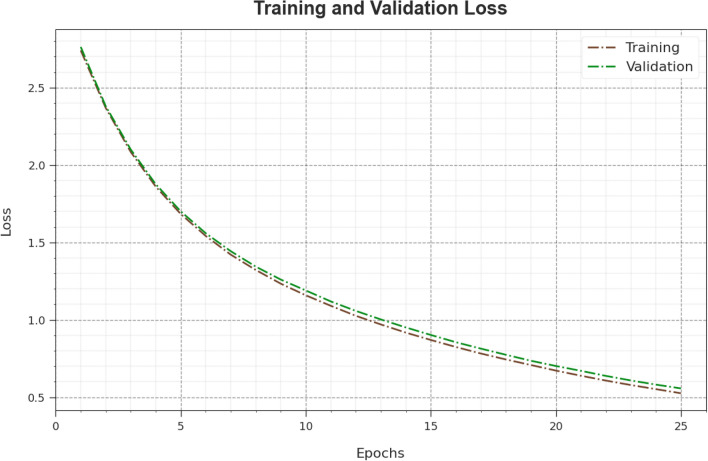
Loss curve of the WSOODL-UAVCSC system.

In Fig. 7, the $TR\_loss$ and $VR\_loss$ effects of the WSOODL-UAVCSC method are exposed. The $TR\_loss$ determined the error between the predicted performance and original values on the TR dataset. The $VR\_loss$ signify the estimation of the performance of the WSOODL-UAVCSC approach on a separate validation dataset. The outcomes denoted that the $TR\_loss$ and $VR\_loss$ tend to reduce with increasing epochs. It depicted the greater performance of the WSOODL-UAVCSC system and its proficiency to produce an accurate classification. The diminished value of $TR\_loss$ and $VR\_loss$ exhibits the improved performance of the WSOODL-UAVCSC procedure on capturing patterns and relationships.

**Figure 8:**
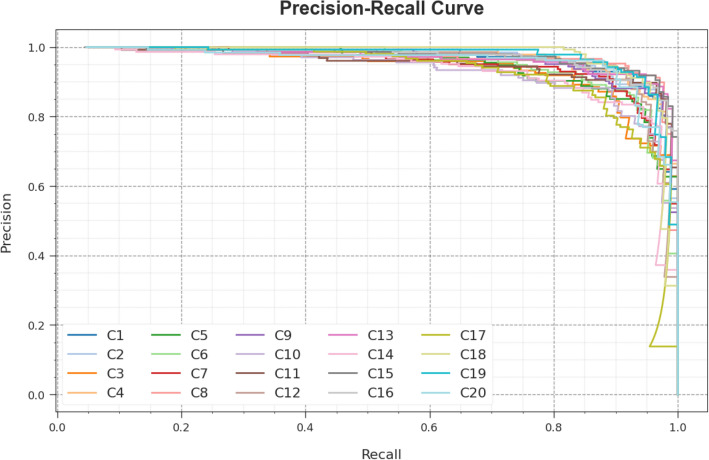
PR curve of the WSOODL-UAVCSC system.

A short precision-recall (PR) analysis of the WSOODL-UAVCSC system is established on the test database in Fig. 8. The outcomes stated that the WSOODL-UAVCSC system outcomes in maximum values of PR. Furthermore, it is perceptible that the WSOODL-UAVCSC approach can achieve greater PR values on all class labels.

**Figure 9:**
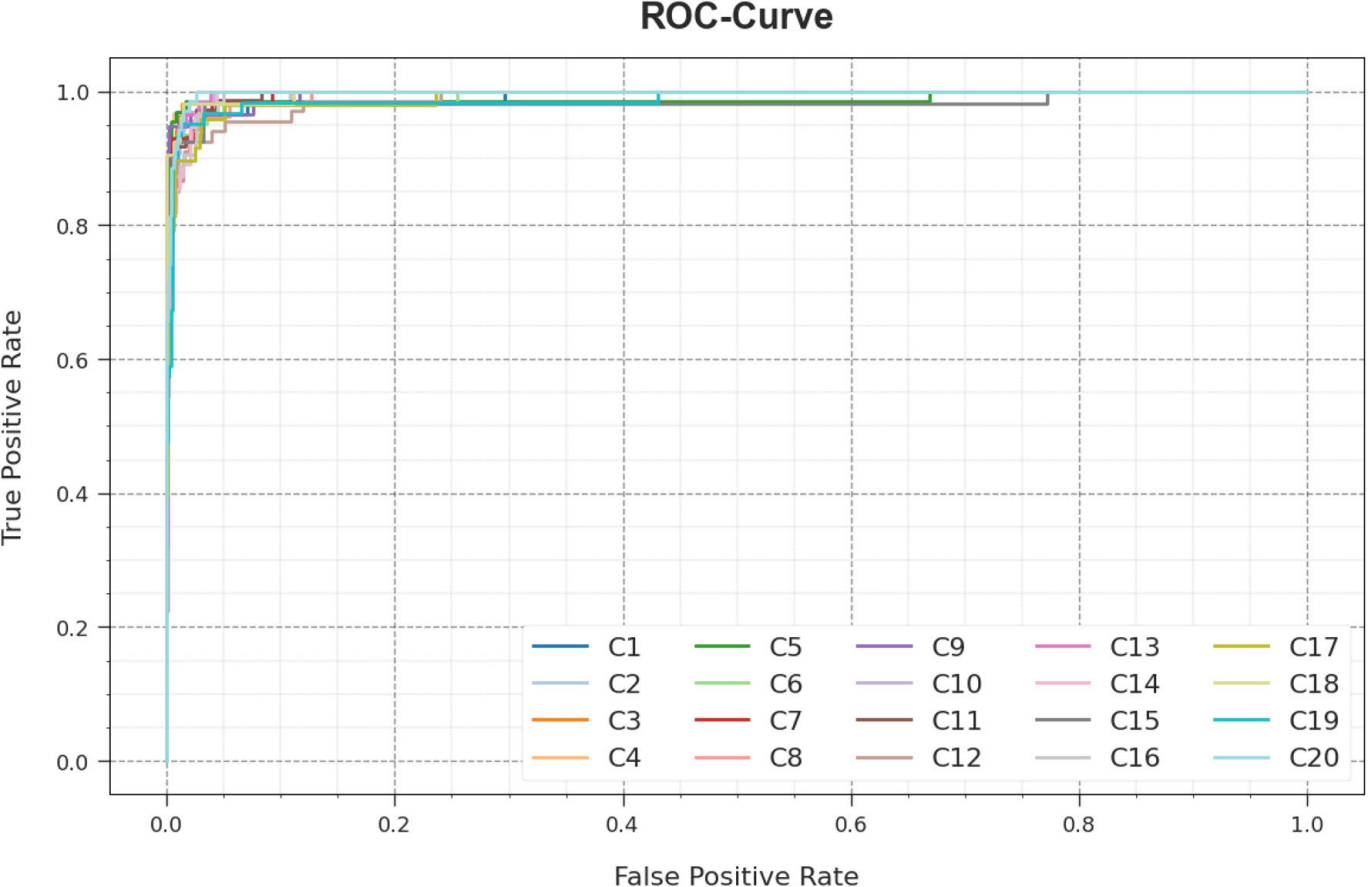
ROC curve of the WSOODL-UAVCSC system.

In Fig. 9, a ROC investigation of the WSOODL-UAVCSC model is shown on the test dataset. The figure defined that the WSOODL-UAVCSC method resulted in the enhancement of ROC values. Additionally, the WSOODL-UAVCSC system can increase ROC values on all class labels.

**Figure 10:**
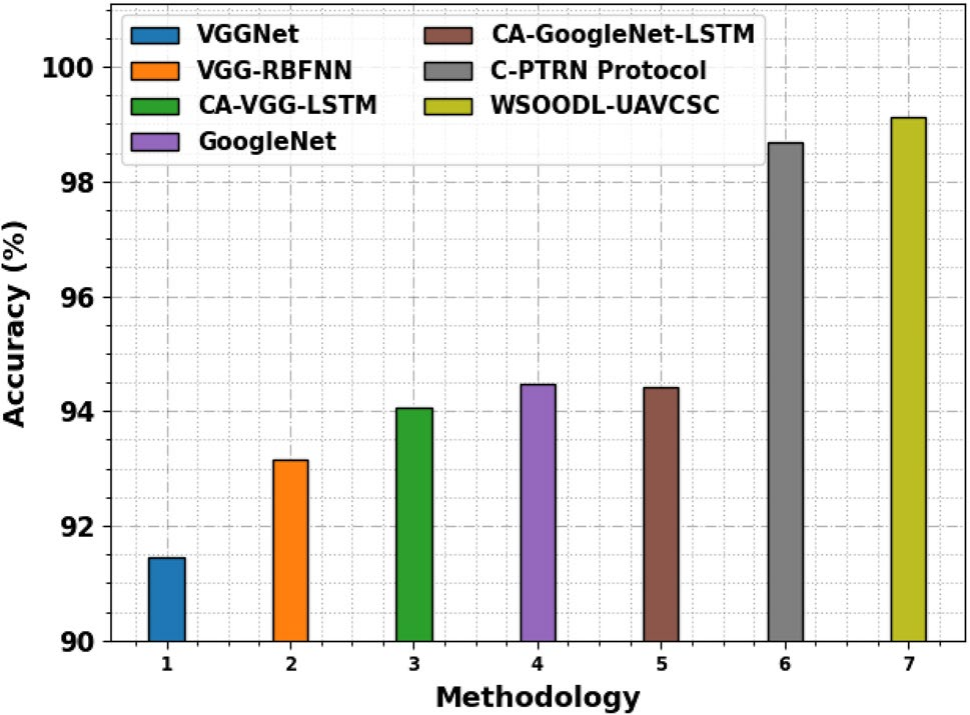
Comparative outcome of WSOODL-UAVCSC system with other methods.

Table 4 and Fig. 10 inspect the scene classification results of the WSOODL-UAVCSC technique with other recent models^10^. The experimental values highlighted that the VGGNet, VGG-RBFNN, CA-VGG-LSTM, GoogleNet, and CA-GoogleNet-LSTM models have obtained poor performance over other models. Simultaneously, the C-PTRN method has shown slightly improved results with $acc{u}_{y}$, $pre{c}_{n}$, $rec{a}_{l}$, and ${F}_{score}$ of 98.67%, 91.65%, 97.45%, and 93.26% respectively. However, the WSOODL-UAVCSC technique gains maximum performance with $acc{u}_{y}$, $pre{c}_{n}$, $rec{a}_{l}$, and ${F}_{score}$ of 99.12%, 97.45%, 98.90%, and 98.10% correspondingly.

**Table 4:** Comparative outcome of WSOODL-UAVCSC system with other methods.

Methodology	Accuracy	Precision	Recall	F-score
VGGNet	91.44	77.77	81.00	78.09
VGG-RBFNN	93.15	79.06	83.90	79.06
CA-VGG-LSTM	94.07	80.68	81.64	80.35
GoogleNet	94.48	81.64	81.97	80.35
CA-GoogleNet-LSTM	94.43	79.06	87.13	81.97
C-PTRN Protocol	98.67	91.65	97.45	93.26
WSOODL-UAVCSC	99.12	97.45	98.90	98.10

**Figure 11:**
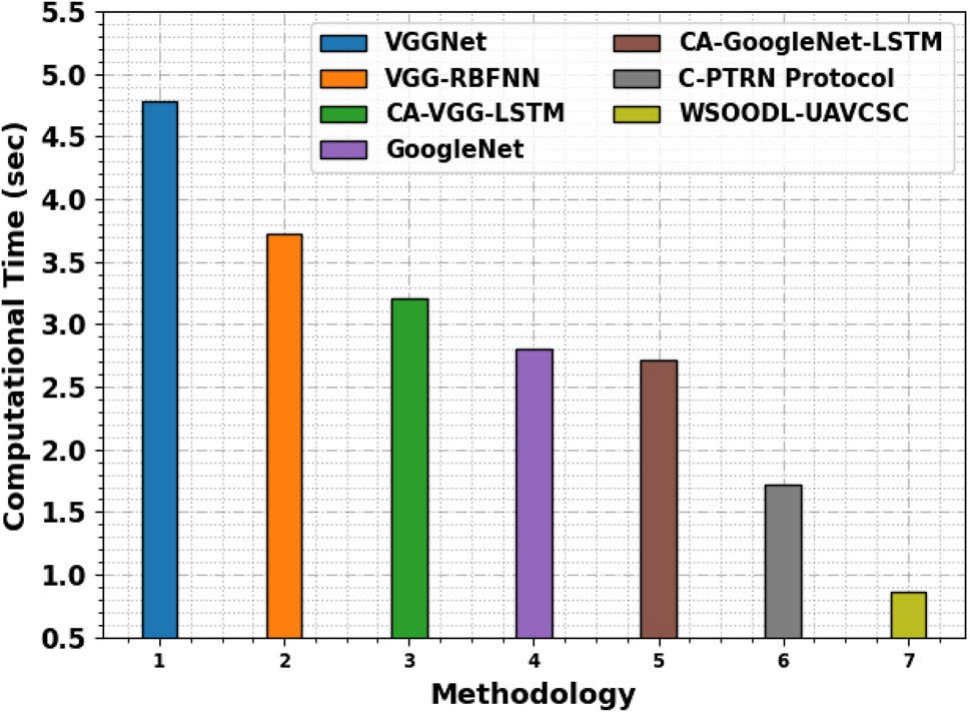
CT outcome of WSOODL-UAVCSC system with other methods.

The CT results of the WSOODL-UAVCSC technique are compared with recent models in Table 5 and Fig. 11. The results indicate that the VGGNet, VGG-RBFNN, CA-VGG-LSTM, GoogleNet, and CA-GoogleNet-LSTM have offered maximum CT values. Next, the C-PTRN model exhibits considerable outcomes with a CT of 1.72s. Nevertheless, the WSOODL-UAVCSC technique offers superior results with the least CT of 0.87s. These results show the betterment of the WSOODL-UAVCSC technique over other models.

**Table 5:** CT outcome of WSOODL-UAVCSC system with other methods.

Methodology	Computational time (s)
VGGNet	4.78
VGG-RBFNN	3.72
CA-VGG-LSTM	3.20
GoogleNet	2.80
CA-GoogleNet-LSTM	2.72
C-PTRN Protocol	1.72
WSOODL-UAVCSC	0.87

## Conclusion

This paper emphasises on the advancement of the WSOODL-UAVCSC system, aiming to enhance transmission efficiency and scene classification within the UAV network. The primary objective of the WSOODL-UAVCSC technique is to effectively cluster UAVs in order to optimise transmission and enhance scene classification. The WSOODL-UAVCSC approach comprises two primary constituents, namely UAV clustering and scene classification. The utilisation of the WSO algorithm in the optimisation of the UAV clustering process facilitates the achievement of efficient communication and interaction within the network. The performance and robustness of the UAV system are enhanced through the utilisation of the WSO method, which incorporates dynamic modification of clustering. The picture classification process incorporates the WSOODL-UAVCSC technique, which encompasses CapsNet feature extraction and classification using ESN. A comprehensive simulation analysis was conducted to verify the superior performance of the WSOODL-UAVCSC approach. The comprehensive analysis of the results revealed that the WSOODL-UAVCSC method exhibited superior performance compared to other current approaches. The suggested model has a possible drawback in its susceptibility to variations in hyperparameter configurations, a concern particularly relevant to deep learning architectures such as CapsNet and ESN. Achieving optimal hyperparameter tuning often requires thorough experimentation and dependence on domain-specific expertise. The validation of the method's effectiveness in real-world UAV applications should be undertaken through the implementation of field testing and trials in future research endeavours. The execution of trials in practical settings including UAV communication and scene classification situations will provide significant knowledge and feedback, hence helping subsequent improvements.

In the future, enhancing the interpretability and explainability of deep learning models utilised for scene categorization and navigation could potentially foster greater trust and acceptance of these methodologies in safety–critical applications. Consequently, this may result in a heightened adoption of these techniques. The examination of novel methodologies for visualising decision-making processes inside these models has the potential to yield UAV systems that exhibit increased transparency and accountability.

## Data Availability

The data that support the findings of this study are available from the corresponding author, upon reasonable request.

## References

[1] Althobaiti A, Alotaibi AA, Abdel-Khalek S, Alsuhibany SA, Mansour RF (2022). Intelligent deep data analytics-based remote sensing scene classification model. Comput. Mater. Continua.

[2] Madokoro H, Sato K, Shimoi N (2019). Vision-based indoor scene recognition from time-series aerial images obtained using a MAV mounted monocular camera. Drones.

[3] Ahmed A, Jalal A, Kim K (2020). A novel statistical method for scene classification based on multi-object categorization and logistic regression. Sensors.

[4] Xu, J., Li, Y., Shi, Q., & He, L., Occluded scene classification via cascade supervised contrastive learning. *IEEE J. Sel. Top. Appl. Earth Observ. Remote Sensing* (2023).

[5] Zheng W, Mo Z, Zhao G (2021). Clustering by errors: a self-organized multitask learning method for acoustic scene classification. Sensors.

[6] Wang F, Qiao J, Li L, Liu Y, Wei L (2022). Scene recognition of road traffic accidents based on an improved faster R-CNN algorithm. Int. J. Crashworthin..

[7] Zou K, Zhao S, Jiang Z (2022). Power line scene recognition based on convolutional capsule network with image enhancement. Electronics.

[8] Zhao, Q., Lyu, S., Li, Y., Ma, Y., & Chen, L. MGML: Multigranularity multilevel feature ensemble network for remote sensing scene classification*. IEEE Trans. Neural Netw. Learn. Syst.* (2021)*.* 10.1109/TNNLS.2021.3106391 34469317

[9] Huang, Y., Cao, X., Zhang, B., Zheng, J., & Kong, X. April. Batch loss regularization in deep learning method for aerial scene classification. In *2017 Integrated Communications, Navigation and Surveillance Conference (ICNS)*, 3E2–1. IEEE (2017).

[10] Neogi N, Bhattacharyya S, Griessler D, Kiran H, Carvalho M (2021). Assuring intelligent systems: Contingency management for UAS. IEEE Trans. Intell. Transp. Syst..

[11] Amir B, Steve P, Souma C (2019). Learning reciprocal actions for cooperative collision avoidance in quadrotor unmanned aerial vehicles. Robot. Auton. Syst..

[12] Li K, Ni W, Dressler F (2021). LSTM-characterized deep reinforcement learning for continuous flight control and resource allocation in UAV-assisted sensor network. IEEE Internet Things J.

[13] Abhik S, Sindhu P, Shalabh B (2021). Memory-based deep reinforcement learning for obstacle avoidance in UAV with limited environment knowledge. IEEE Trans. Intell. Transp. Syst..

[14] Pi CH, Dai YW, Hu KC, Cheng S (2021). General purpose low-level reinforcement learning control for multi-axis rotor aerial vehicles. Sensors.

[15] Yuichi K, Hiroki N, Nei K, Fumie O, Ryu M (2019). Toward future unmanned aerial vehicle networks: Architecture, resource allocation and field experiments. IEEE Wirel. Commun..

[16] Phuong L, Francois G, Le-Nam T, Fabrice L (2021). Deep reinforcement learning-based resource allocation in cooperative UAV-assisted wireless networks. IEEE Trans. Wirel. Commun..

[17] Oubbati OS, Lakas A, Lorenz P, Atiquzzaman M, Jamalipour A (2021). Leveraging communicating UAVs for emergency vehicle guidance in urban areas. IEEE Trans. Emerg. Top. Comput..

[18] Vuk M, Ismail G, Rudra D, Mihail LS, Brian F (2020). Advanced wireless for unmanned aerial systems: 5G Standardization, research challenges, and AERPAW architecture. IEEE Veh. Technol. Mag..

[19] Florence H, Ruben G, Artur G, Bastien R, Benjamin S, Daisuke K, Marc C, Helmut P (2020). Decentralized multi-agent path finding for UAV traffic management. IEEE Trans. Intell. Transp. Syst..

[20] Duncan BA, Murphy RR (2014). Autonomous capabilities for small unmanned aerial systems conducting radiological response: Findings from a high-fidelity discovery experiment. J. Field Robot..

[21] Martínez-Vargas A, Rodríguez-Cortés GL, Montiel-Ross O (2019). Comparative representations of a genetic algorithm to locate unmanned aerial vehicles in disaster zones. Eng. Lett..

[22] Sihem O, Miloud B, Jonathan P-G, Tarik T (2021). Deep reinforcement learning based collision avoidance in UAV environment. IEEE Internet Things J..

[23] Girma A, Bahadori N, Sarkar M, Tadewos TG, Behnia MR, Mahmoud MN, Karimoddini A (2020). IoT-enabled autonomous system collaboration for disaster-area management. IEEE/CAA J. Autom. Sin..

[24] Masaracchia A, Li Y, Nguyen KK, Yin C, Khosravirad SR, Da Costa DB, Duong TQ (2021). UAV-enabled ultra-reliable low-latency communications for 6g: a comprehensive survey. IEEE Access.

[25] Pustokhina IV, Pustokhin DA, Kumar Pareek P, Gupta D, Khanna A, Shankar K (2021). Energy-efficient cluster-based unmanned aerial vehicle networks with deep learning-based scene classification model. Int. J. Commun. Syst..

[26] Rajagopal A, Joshi GP, Ramachandran A, Subhalakshmi RT, Khari M, Jha S, Shankar K, You J (2020). A deep learning model based on multi-objective particle swarm optimization for scene classification in unmanned aerial vehicles. IEEE Access.

[27] Li H, Shi Y, Zhang B, Wang Y (2018). Superpixel-based feature for aerial image scene recognition. Sensors.

[28] Tong G, Nan J, Biyue Li, Zhu Xi, Ya W, Wenbo Du (2021). UAV navigation in high dynamic environments: A deep reinforcement learning approach. Chin. J. Aeronaut..

[29] Uthayan, K.R., Prasad, G.L.V., Mohan, V., Bharatiraja, C., Pustokhina, I.V., Pustokhin, D.A. and García Díaz, V. IoT-cloud-empowered aerial scene classification for unmanned aerial vehicles. Comput. Mater. Continua (2022)

[30] Li Z, Zhou A (2021). Self-selection salient region-based scene recognition using slight-weight convolutional neural network. J. Intell. Robot. Syst..

[31] Xia J, Ding Y, Tan L (2021). Urban remote sensing scene recognition based on lightweight convolution neural network. IEEE Access.

[32] Ming C, Zhiye W, Yiting T, Jiangbo Xi, Chaofeng R, Miaozhong Xu (2021). Unsupervised self-adaptive deep learning classification network based on the optic nerve microsaccade mechanism for unmanned aerial vehicle remote sensing image classification. Geocarto Int..

[33] Nilakshi D, Bhogeswar B (2021). A novel mutual information-based feature selection approach forefficient transfer learning in aerial scene classification. Int. J. Remote Sens..

[34] Yu Z, Jifeng G, Chengchao B, Hongxing Z (2021). Reinforcement learning-based collision avoidance guidance algorithm for fixed-wing UAVs. Complexity.

[35] Yu-Hsin H, Rung-Hung G (2020). Reinforcement learning-based collision avoidance and optimal trajectory planning in UAV communication networks. IEEE Trans. Mobile Comput..

[36] Oualid D, Deok JL (2021). Deep reinforcement learning for end-to-end local motion planning of autonomous aerial robots in unknown outdoor environments: real-time flight experiments. Sensors.

[37] Chao W, Jing W, Jingjing W, Xudong Z (2020). Deep-reinforcement-learning-based autonomous UAV navigation with sparse rewards. IEEE Internet Things J..

[38] Liu CH, Ma X, Gao X, Tang J (2020). Distributed energy-efficient multi-uav navigation for long-term communication coverage by deep reinforcement learning. IEEE Trans. Mobile Comput..

[39] Sampedro C, Rodriguez-Ramos A, Bavle H, Carrio A, de la Puente P, Campoy P (2019). A fully-autonomous aerial robot for search and rescue applications in indoor environments using learning-based techniques. J. Intell. Robot. Syst..

[40] Hang Qi, Zhiqun Hu, Hao H, Xiangming W, Zhaoming Lu (2020). Energy efficient 3-D UAV control for persistent communication service and fairness: A deep reinforcement learning approach. IEEE Access.

[41] Ben Aissa S, Ben Letaifa A (2021). UAV communications with machine learning: Challenges, applications and open issues. Arab. J. Sci. Eng..

[42] Jiseon M, Savvas P, Christos L, Panayiotis K, Sunwoo K (2021). Deep reinforcement learning multi-UAV trajectory control for target tracking. IEEE Internet Things J..

[43] Chao Y, Chang W, Xiaojia X, Lan Z, Yuna J (2022). Deep reinforcement learning of collision-free flocking policies for multiple fixed-wing UAVs using local situation maps. IEEE Trans. Indust. Inform..

[44] Alhumade H, Rezk H, Louzazni M, Moujdin IA, Al-Shahrani S (2023). Advanced energy management strategy of photovoltaic/PEMFC/lithium-ion batteries/supercapacitors hybrid renewable power system using white shark optimizer. Sensors.

[45] Alsolai H, Alzahrani JS, Maray M, Alghamdi M, Qahmash A, Alnfiai MM, Aziz ASA, Mustafa Hilal A (2022). Enhanced artificial gorilla troops optimizer-based clustering protocol for UAV-assisted intelligent vehicular network. Drones.

[46] Saravagi, D., Agrawal, S., Saravagi, M., Jain, S.K., Sharma, B., Mehbodniya, A., Chowdhury, S. & Webber, J.L., Predicting lumbar spondylolisthesis: A hybrid deep learning approach.

[47] Zaki MM, Chen S, Zhang J, Feng F, Qi L, Mahdy MA, Jin L (2023). Optimized weighted ensemble approach for enhancing gold mineralization prediction. Appl. Sci..

[48] Tan C, Tan W, Shen Y, Yang L (2023). Multistep wind power prediction using time-varying filtered empirical modal decomposition and improved adaptive sparrow search algorithm-optimized phase space reconstruction-echo state network. Sustainability.

[49] http://weegee.vision.ucmerced.edu/datasets/landuse.html

[50] Sarfraz DA (2022). A novel ensemble learning method using multiple objective particle swarm optimization for subject-independent EEG-based emotion recognition. Comput. Biol. Med..

[51] Omurkanova AT (2022). A new brain tumor diagnostic model: Selection of textural feature extraction algorithms and convolution neural network features with optimization algorithms. Comput. Biol. Med..

[52] Mohammad-Hossein N-S, Hoda Z, Seyedali M (2022). Enhanced whale optimization algorithm for medical feature selection: A COVID-19 case study. Comput. Biol. Med..

[53] Kappelhof N, Ramos LA, Kappelhof M, van Os HJ, Chalos V, van Kranendonk KR, Kruyt ND, Roos YB, van Zwam WH, van der Schaaf IC, van Walderveen MA (2021). Evolutionary algorithms and decision trees for predicting poor outcome after endovascular treatment for acute ischemic stroke. Comput. Biol. Med..

[54] Martínez-Río J, Carmona EJ, Cancelas D, Novo J, Ortega M (2021). Robust multimodal registration of fluorescein angiography and optical coherence tomography angiography images using evolutionary algorithms. Comput. Biol. Med..

